# Supplementary material for: Partially Hydrolyzed Guar Gum Suppresses the Development of Sarcopenic Obesity
Source: Nutrients. 2022 Mar 9;14(6):1157. doi: 10.3390/nu14061157 (PMC8955723; doi:10.3390/nu14061157)
Supplement: Supplementary file 1 [file nutrients-14-01157-s001.zip › nutrients-1593794-supplementary.pdf]

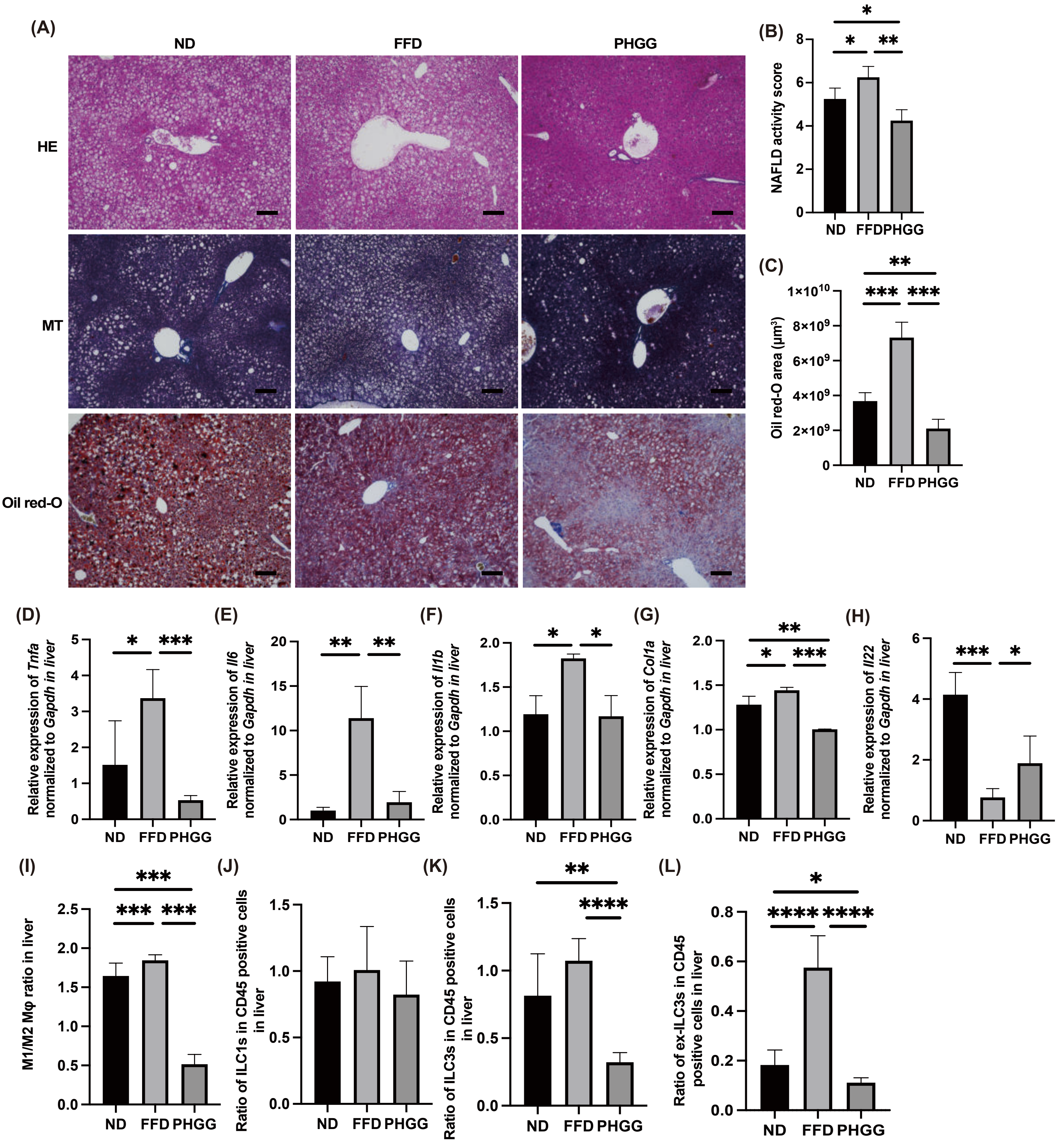
(A)
ND
FFD
PHGG
HE
MT
Oil red-O
(B)
NAFLD activity score
ND FFD PHGG
(C)
Oil red-O area (μm³)
ND FFD PHGG
(D)
Relative expression of Tnfa normalized to Gapdh in liver
(E)
Relative expression of Il6 normalized to Gapdh in liver
(F)
Relative expression of Il1b normalized to Gapdh in liver
(G)
Relative expression of Col1a normalized to Gapdh in liver
(H)
Relative expression of Il22 normalized to Gapdh in liver
(I)
M1/M2 Mφ ratio in liver
(J)
Ratio of ILC1s in CD45 positive cells in liver
(K)
Ratio of ILC3s in CD45 positive cells in liver
(L)
Ratio of ex-ILC3s in CD45 positive cells in liver
ND FFD PHGG

FIgure 2

Supplementary Figure S1. Administration of PHGG improved fatty liver and decreased the expression ofgenes related to inflammation, liver fibrosis, and fatty acid metabolism, and decreased the ratio of M1 macrophages, ILC1, ILC3, and ex-ILC3.

(A) Representative images of hematoxylin & eosin-, Masson trichrome-, and oil red-O- stained liver sections. Liver tissues were collected at 16-weeks of age. The scale bar shows 100 μm.

(B) Nonalcoholic fatty liver disease (NAFLD) activity scores (n = 6). (C) Oil red-O-stained area (n = 6). Relative mRNA expression of (D) Tnfa, (E) Il6, (F) Il1b, (G) Col1a, and (H) Il22 in the liver normalized to the expression of Gapdh (n = 6). Ratio of (I) M1 to M2 macrophages, (J) ILC1s to CD45-positive cells, (K) ILC3s to CD45-positive cells, and (L) ex-ILC3s to CD45-positive cells in the liver (n = 6 in each case). Data are represented as the mean ± SD values. *p<0.05, **p<0.01, ***p<0.001, **** p < 0.0001, as determinedby one-way ANOVA.

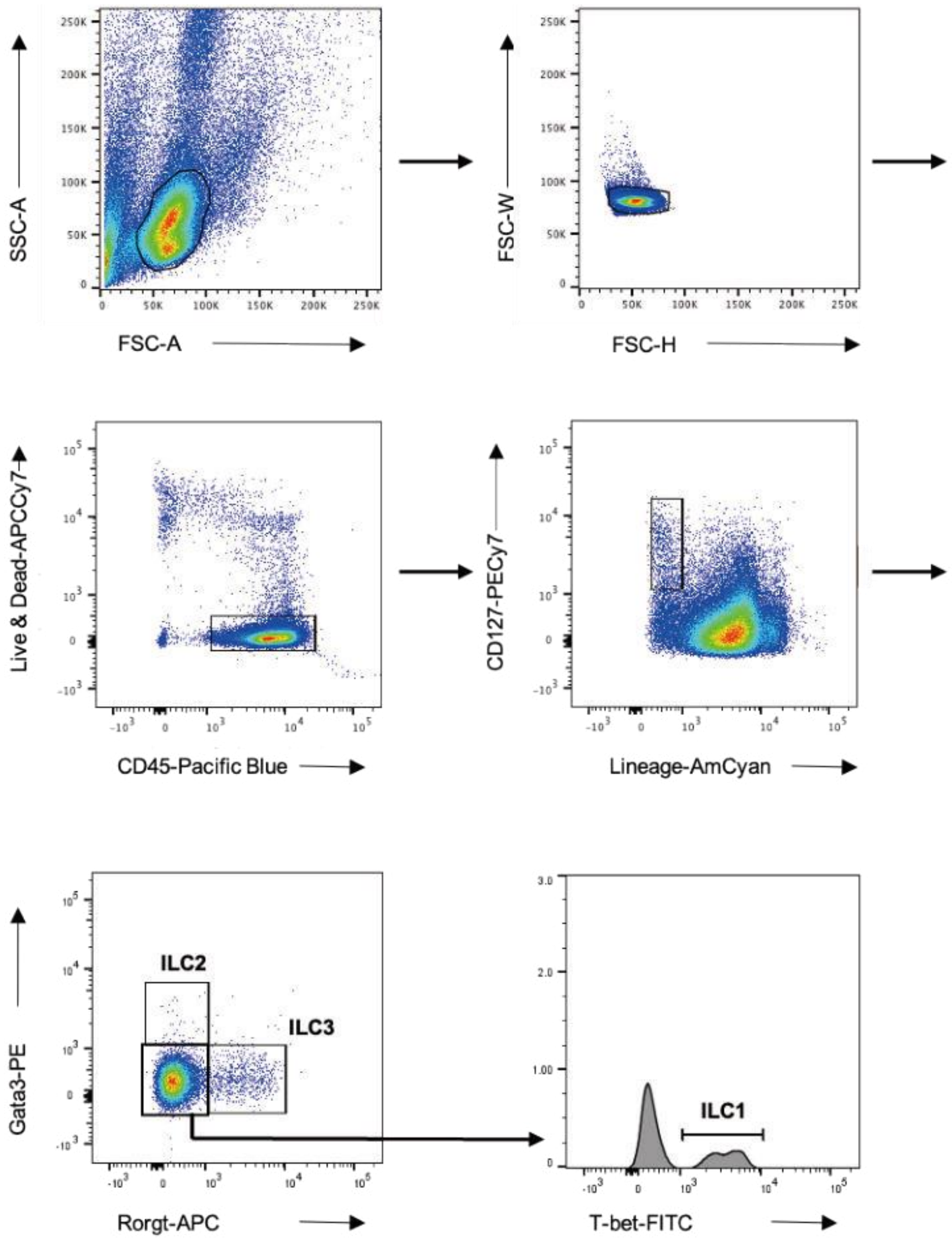

Supplementary Figure S2. Strategy for innate lymphoid cells (ILCs)

Representative flow cytometry plots of liver CD45+ Live & Dead- Lin- CD127+ RORg- GATA-3- T-bet+ ILC1s, CD45+ Live & Dead- Lin- CD127+ RORg- GATA-3+ ILC2s and CD45+ Live & Dead- Lin- CD127+ RORg+ GATA-3- ILC3s in each group at 16-weeks of age.

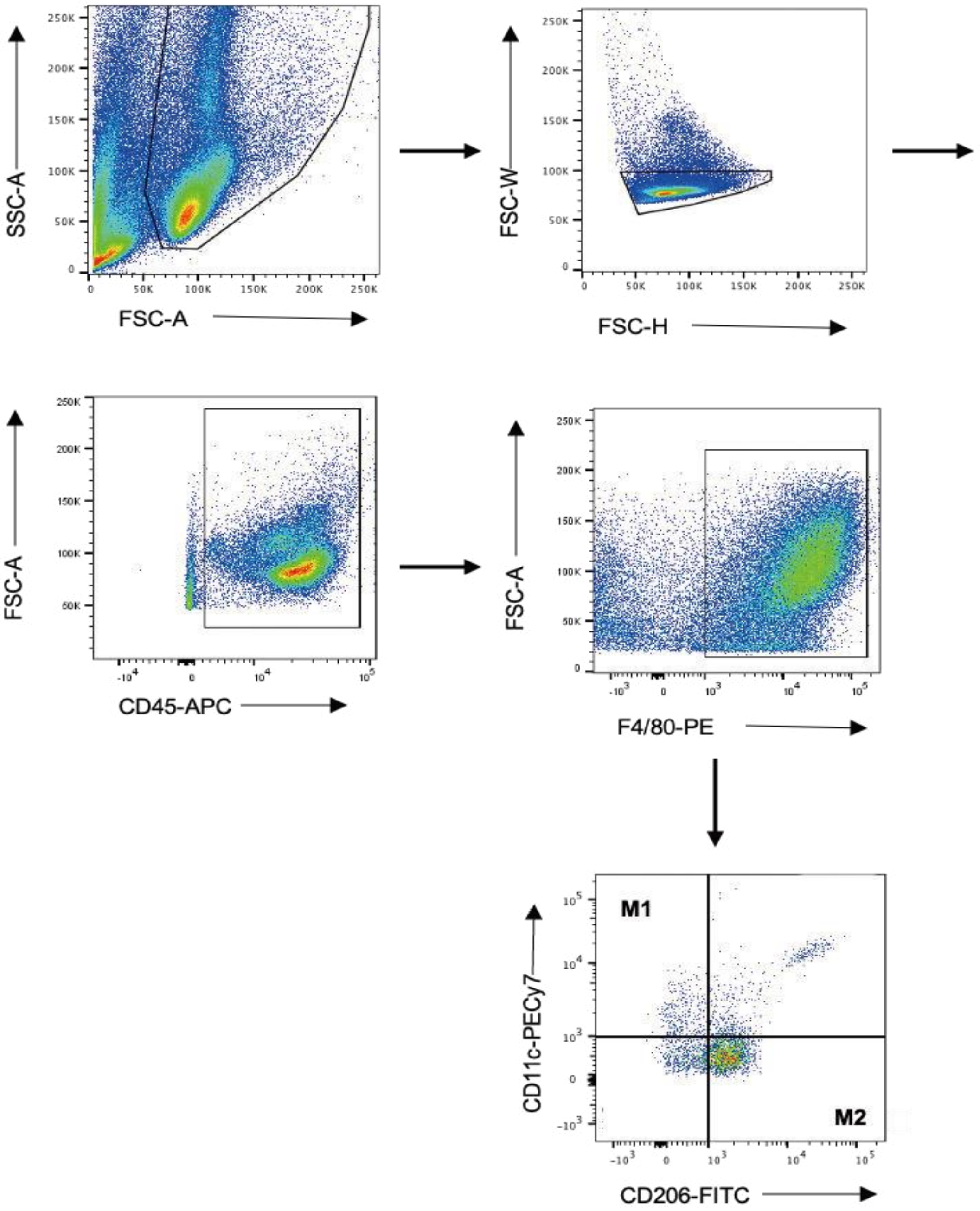

Supplementary Figure S3. Strategy for macrophages

Representative flow cytometry plots of liver CD45+ F4/80+ CD206- CD11c+ M1 macrophages and CD45+ F4/80+ CD206+ CD11c- M2 macrophages in each group at 16 weeks of age.

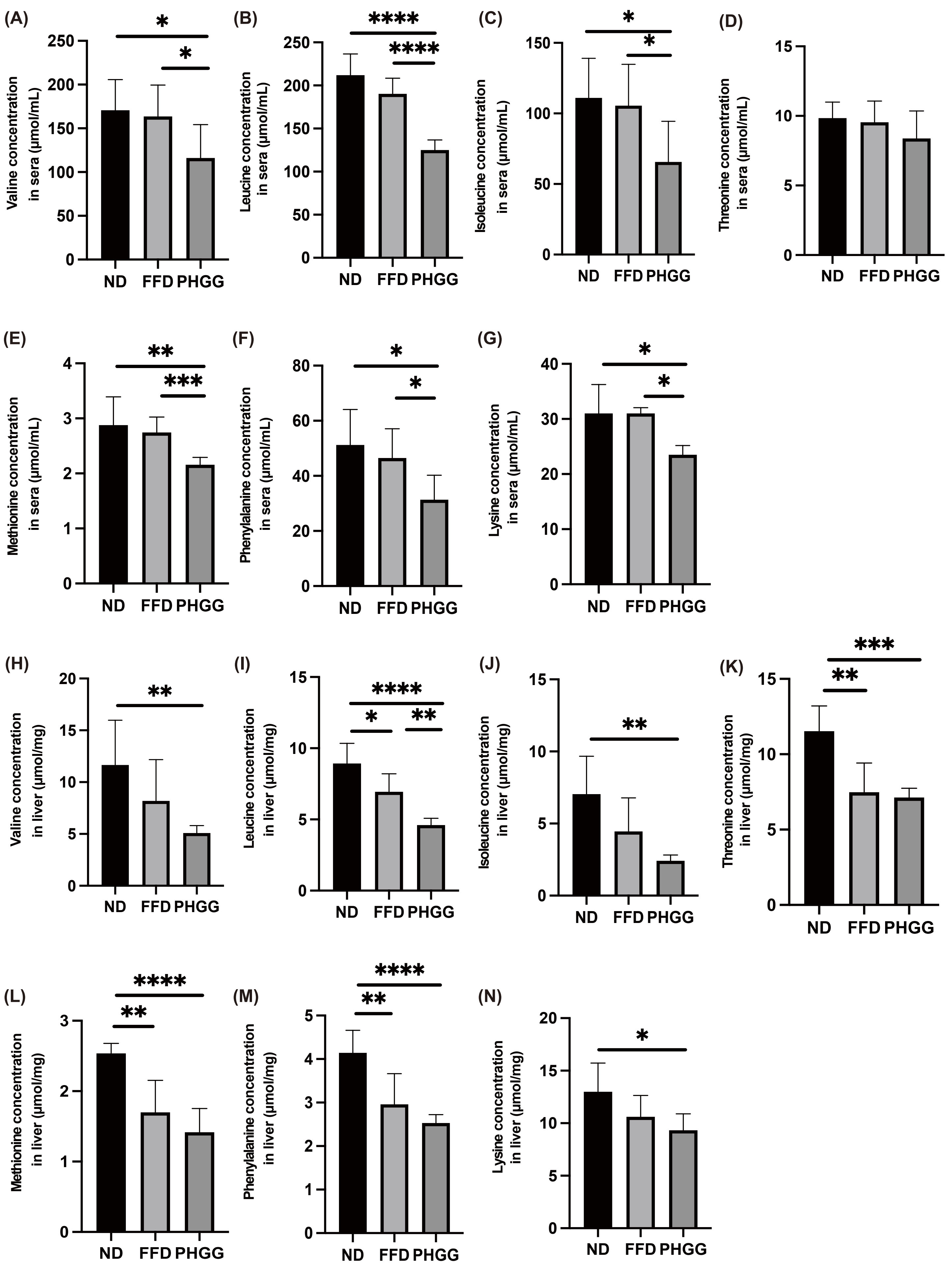
(A)
Valine concentration in sera (μmol/mL)
(B)
Leucine concentration in sera (μmol/mL)
(C)
Isoleucine concentration in sera (μmol/mL)
(D)
Threonine concentration in sera (μmol/mL)
(E)
Methionine concentration in sera (μmol/mL)
(F)
Phenylalanine concentration in sera (μmol/mL)
(G)
Lysine concentration in sera (μmol/mL)
(H)
Valine concentration in liver (μmol/mg)
(I)
Leucine concentration in liver (μmol/mg)
(J)
Isoleucine concentration in liver (μmol/mg)
(K)
Threonine concentration in liver (μmol/mg)
(L)
Methionine concentration in liver (μmol/mg)
(M)
Phenylalanine concentration in liver (μmol/mg)
(N)
Lysine concentration in liver (μmol/mg)
ND
FFD
PHGG

Supplementary Figure S4. The administration of PHGG decreased the concentration of the amino acidsrelated to muscle biosynthesis.

The concentrations of (A) valine, (B) leucine, (C) isoleucine, (D) threonine, (E) methionine, (F) phenylalanine, and (G) lysine in the sera ($n = 6$). The concentrations of (H) valine, (I) leucine, (J) isoleucine, (K) threonine, (L) methionine, (M) phenylalanine, and (N) lysine in the liver ($n = 6$).

Data are represented as the mean ± SD values; *$p < 0.05$, **$p < 0.01$, ***$p < 0.001$, **** $p < 0.0001$, as determinedby one-way ANOVA.

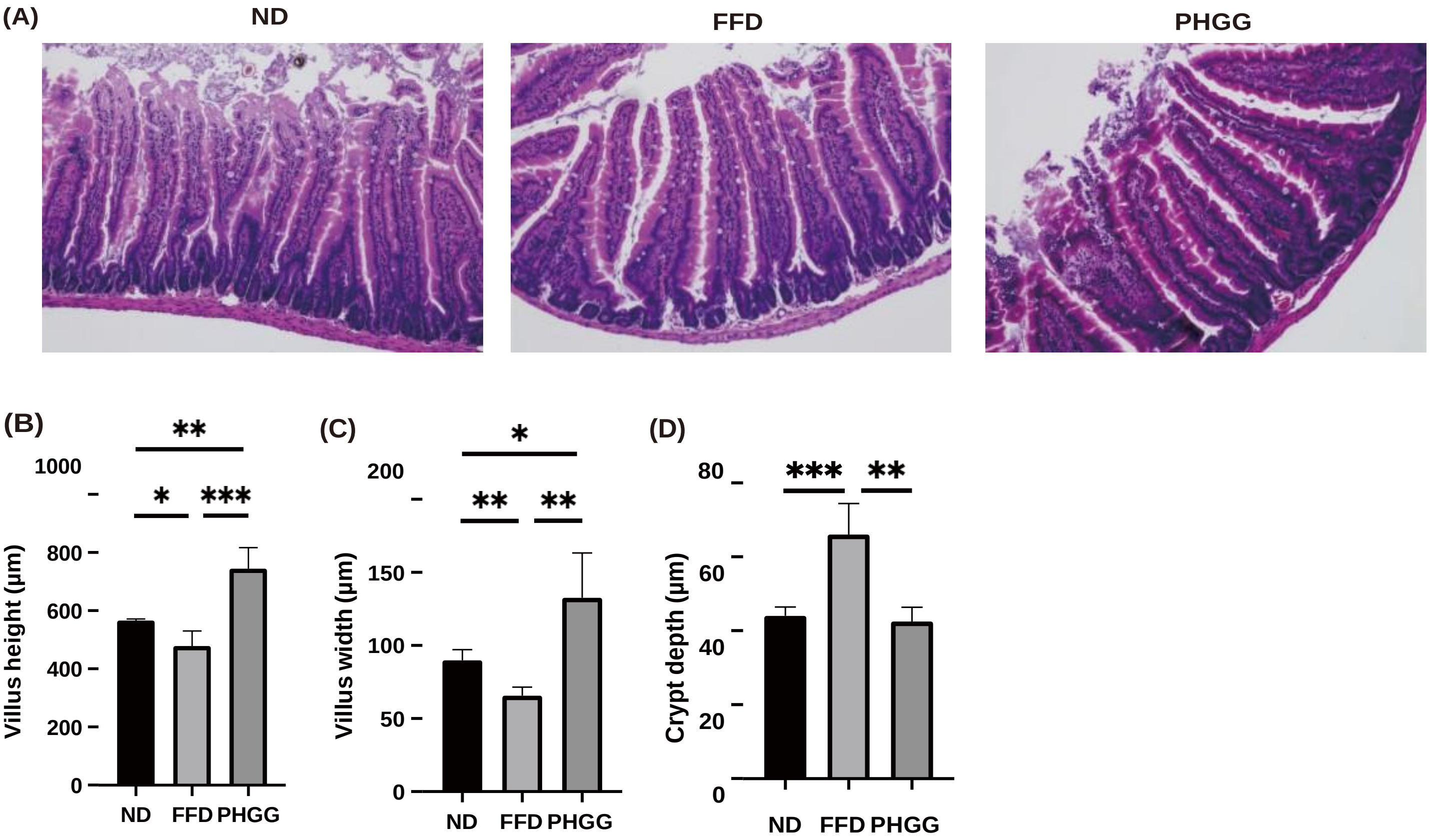

Supplementary Figure S5. Administratio of PHGG reduced small intestinal inflammation.

(A) Representative images showing the histological features of the jejunum of the mice.

(B) Villus height of the jejunum (n = 6). (C) Villus width of the jejunum (n = 6). (D) Crypt depth of the jejunum (n = 6).

Scale bar: 100μm.

Data have been represented in terms of mean ± SD values. *p < 0.05, **p < 0.01, and ***p < 0.001 using a paired t-test.
